# Prognosis of Unresectable Hepatocellular Carcinoma: Comparison of Seven Staging Systems (TNM, Okuda, BCLC, CLIP, CUPI, JIS, CIS) in a Chinese Cohort

**DOI:** 10.1371/journal.pone.0088182

**Published:** 2014-03-07

**Authors:** Jin-feng Zhang, Zhi-jun Shu, Chun-yi Xie, Qi Li, Xi-hong Jin, Wei Gu, Fang-jian Jiang, Chang-quan Ling

**Affiliations:** 1 Department of Traditional Chinese Medicine, Shanghai Hospital of Chinese Integrative Medicine, Shanghai, China; 2 Department of Surgery, Shanghai Hospital of Chinese Integrative Medicine, Shanghai, China; 3 Department of Cardiology, Shanghai Hospital of Chinese Integrative Medicine, Shanghai, China; 4 Department of Medical Oncology, Shuguang Hospital, Shanghai University of Traditional Chinese Medicine, Shanghai, China; 5 Department of Massage, Yueyang Hospital of Integrated Chinese and Western Medicine, Shanghai University of Traditional Chinese Medicine, Shanghai, China; 6 Department of Traditional Chinese Medicine, Changhai Hospital, Second Military Medical University, Shanghai, China; University of North Carolina School of Medicine, United States of America

## Abstract

**Background:**

Many liver staging systems that include the tumor stage and the extent of liver function have been developed. However, prognosis assessment for hepatocellular carcinoma (HCC) remains controversial. In this study, the performances of 7 staging systems were compared in a cohort of patients with HCC who underwent non-surgical treatment.

**Methods:**

A total of 196 consecutive patients with HCC who underwent non-surgical treatment seen between January 1, 2004, and December 31, 2007, were included. Performances of TNM sixth edition, Okuda, Barcelona Clinic Liver Cancer (BCLC), Cancer of the Liver Italian Program (CLIP), Chinese University Prognostic Index (CUPI), Japan Integrated Staging (JIS), and China integrated score (CIS) have been compared and ranked using concordance index (c-index). Predictors of survival were identified using univariate and multivariate Cox model analyses.

**Results:**

The median survival time for the cohort was 7.6 months (95% CI 5.6–9.7). The independent predictors of survival were performance status (*P*<.001), serum sodium (*P*<.001), alkaline phosphatase (*P*<.001), tumor diameter greater than 5 cm (*P* = .001), portal vein invasion (*P*<.001), lymph node metastasis (*P* = .025), and distant metastasis (*P* = .004). CUPI staging system had the best independent predictive power for survival when compared with the other six prognostic systems. Performance status and serum sodium improved the discriminatory ability of CUPI.

**Conclusion:**

In our selected patient population whose main etiology is hepatitis B, CUPI was the most suitable staging system in predicting survival in patients with unresectable HCC. BCLC was the second top-ranking staging system. CLIP, JIS, CIS, and TNM sixth edition were not helpful in predicting survival outcome, and their use is not supported by our data.

## Introduction

Clinical staging for cancers provides a guidance to predict survival outcome and to decide optimal treatment strategies. Whereas, unlike other solid tumors, the prognosis and treatment options for patients with hepatocellular carcinoma (HCC) depend not only on the tumor stage but also on the extent of liver dysfunction. On the basis of identification of relevant prognostic factors for both the liver cancer and liver function, many staging systems that included both aspects had been developed.

The mainly reported staging systems include TNM sixth edition [1], Okuda [2], Barcelona Clinic Liver Cancer (BCLC) [3], Cancer of the Liver Italian Program (CLIP) [4], [5], Chinese University Prognostic Index (CUPI) [6], [7], Japan Integrated Staging (JIS) [8], [9], and China integrated score (CIS) [10].

CIS was a new staging system recently proposed by Bai-Hong Zhang *et al* for the patients with unresectable HCC. As limited numbers of HCC patients are eligible for curative therapies such as surgery or ablation in Asia at present. So far, it lacks of a quantitative assessment of its predictive value.

Other staging systems (except for CIS staging) have been compared and ranked in different studies [11]–[17] according to their prognostic value. Results were not consistent between studies. Which staging system was best remained controversial. This can probably be partly explained by the difference of the characteristics in the investigated populations of different disease stage and by different etiology.

This study focuses on patients with unresectable HCC in an intermediate-advanced setting mainly associated with hepatitis B virus etiology. We have assessed and compared the performance of seven prognostic classifications (TNM sixth edition, Okuda, BCLC, CLIP, CUPI, JIS and CIS) for predicting overall survival. We also explore whether the best staging systems could be improved by adding other clinical or biological variables not included in these systems.

## Patients and Methods

### Patients

The study protocol was approved by the institutional ethnic committee at the Changhai Hospital of the Second Military Medical University. Written informed consent was given by participants for their clinical records to be used in the study. The 201 consecutive patients with unresectable HCC at the Department of Gastroenterology of Changhai Hospital between January 1, 2004, and December 31, 2007 were retrospectively identified by medical oncologist. 5 patients were lost to follow up. Finally, 196 patients were entered in the study. The diagnosis of HCC was verified histologically by percutaneous needle biopsy in 3 patients. Other patients were diagnosed on the basis of radiologic criteria according to European Association for the Study of the Liver - two imaging studies (computed tomography [CT] or magnetic resonance imaging [MRI]) showing an arterial enhancing mass greater than 2 cm, or one imaging study (CT or MRI) showing an arterial enhancing mass greater than 2 cm and an alpha-fetoprotein (AFP) greater than 400 ng/mL [18].

A baseline evaluation including clinical examination, laboratory studies, and imaging studies (CT or MRI) was performed. The survival time was defined as from the date of diagnosis to the date of death or last contact for surviving patients. The study was censored on January 1, 2010.

Unresectable HCC was defined as a liver tumor not eligible for resection therapy given the extent of disease, including patients that were not suitable for surgery for location of tumor(s) in the liver, or patients who were older than 75 years, or those who refused surgical therapies. Liver transplantation is still rare in China.

According to tumor characteristics and liver functional status, patients with unresectable HCC underwent locoregional therapy including percutaneous ethanol injection, or transarterial chemoembolization, or best supportive care. Only a few of patients underwent percutaneous ethanol injection. Typically, such patients are considered candidates for transarterial chemoembolization and best supportive care.

Patients were excluded if any data relative to the seven classifications considered were missing or if there was no available follow-up information for a retrospective prognostic analysis. Patients who could not undergo surgery for existing chronic extrahepatic diseases were also excluded.

### Data collection

Data needed to stage patients in all seven staging systems and that could be risk factors for developing HCC were retrieve from the electronic medical records. These included a wide range of demographics, clinical, laboratory and imaging data in order to further characterize our HCC-collective.

Specifically, the following variables were collected for the analysis: age and gender of the patient, date of HCC diagnosis and date of death or last information, presence of cirrhosis, etiology, patient' general condition (abdominal pain, weight loss, Eastern Cooperative Oncology Group performance status [ECOG PS]), liver cirrhosis clinical manifestations (ascites, signs of portal hypertension and encephalopathy), main serological parameters (total bilirubin, albumin, alanine aminotransferase [ALT], aspartate aminotransferase [AST], alkaline phosphatase [ALP], gamma glutamyl transpeptidase [γ-GT], blood urea nitrogen, serum creatinine, prothrombin time, alpha-fetoprotein [AFP] levels), tumor characteristics (number of lesions, diameter of largest lesion, lobar involvement, vasclular invasion, portal vein thrombosis, organ invasion, nodes status) and treat type.

Weight loss was defined as more than 7 kg loss in weight within 3 months before presentation. Tumor characteristics were retrospectively recorded from the radiology report. Child-Pugh score that evaluated the hepatic function was calculated from obtained clinical and laboratory data. Cirrhosis was diagnosed by biopsy specimen or unequivocal clinical (gastroesophageal varices, splenomegaly with a platelet count of less than 100, 000/ml, ascites) and radiological evidence of portal hypertension. TNM fifth edition stages of patients were identified to calculate the CUPI scores.

### Staging

All patients assessed were restaged according to the TNM sixth edition, Okuda, BCLC, CLIP, CUPI, JIS, and CIS stage system by collected data.

### Statistical analysis

Overall survival times of studied patients with unresectable HCC were estimated by using the Kaplan-Meier method from the date of diagnosis to the date of death or last follow-up time. Survivals of all Patients were stratified according to prognostic categories for each of the seven staging systems. Differences in survival among their prognostic strata were compared by using the log-rank test. Independent predictors of survival were identified by Cox's proportional hazard model using the stepwise selection of variables. Clinical variables that were of significant prognostic value in univariate analysis were subsequently included in a multivariate analysis.

Staging systems were ranked by using the concordance index (c-index), that measures the discrimination ability of the different staging systems to stratify patients with different outcomes: higher statistic value of the c- index, better the model is about a patient' prognosis. The c- indices derived were compared among the different staging systems by using bootstrap and the conclusions that we reached were tested by applying random resampling.

The prognostic variables not included these staging systems were identified, and then added to the top-ranked staging system. A new value of c-index was generated and resulting model was internally validated by using bootstrap to measure the improvement of resulting model.

## Results

### Patient characteristics and overall survival

A total of 196 patients with unresectable HCC were included in the study. They included 169 male and 27 female patients, with a median age of 56 years (range 22–84 years). Their demographic, clinical, laboratory, tumor characteristics are listed in **Table 1** and **Table 2**. Of the 196 patients, at the time the data were censored, 185 (94.4%) patients died during the study period. The median survival time was 7.6months (95% CI 5.6–9.7), and the median follow-up time was 18.1 (range 0.2–59.7) months. The median overall survival at 1 year and 2 years from admission was 23.3% and 5.6%, respectively.

**Table 1:** Demographic, clinical information of 196 patients with unresectable HCC.

Characteristic	Patients (%)
**Age, years**	
Median	56
Range	22–84
**Sex, %**	
Male	86
Female	14
**Etiology, %**	
Hepatitis B	89
Hepatitis C	3
Alcohol	2
Other	1
Cryptogenic	5
**Cirrhosis, %**	
Yes	81
No	19
**Symptoms, %**	
Present	67
Absent	33
**ECOG PS, %**	
0–1	29
2–3	71
**Abdominal pain, %**	
Yes	55
No	45
**Weight loss, %**	
Yes	34
No	66
**Ascites, %**	
Yes	76
No	24
**Jaundice, %**	
Yes	33
No	67
**Esophageal varices, %**	
Yes	28
No	72
**Encephalopathy, %**	
Yes	3
No	97
**Portal hypertension, %**	
Yes	83
No	17
**Laboratory values, medians**	
Total bilirubin (µmol/l)	22.5
Albumin (g/l)	35
Prothrombin time (s)	14.75
ALT (U/L)	36
AST (U/L)	54
ALP (IU/L)	138
γ-GT (IU/L)	166
Serum sodium (mmol/l)	140
Platelets (K/µL)	135
Blood urea nitrogen (mmol/l)	4.9
Serum creatinine (µmol/l)	72
AFP (µg/l)	246.25
**Tumor characteristic, %**	
Number of lesions	
1–5	44
>5	56
Diameter of largest lesion (cm)	5.2
Lobar involvement	
Unilobar	68
Bilobar	32
Extent	
≤50%	63
>50%	37
Vascular invasion	
Yes	52
No	48
Portal vein thromboses	
Yes	45
No	55
Lymph node metastasis	
Yes	27
No	73
Organ invasion	
Yes	6
No	94
T	
0–2	18
3–4	82
N	
Yes	87
No	13
M	
Yes	15
No	85
**Treatment offered, %**	
PEI+TACE	3
TACE	58
Best support care	39

Abbreviations: HCC: hepatocellular carcinoma; ECOG PS: Eastern Cooperative Oncology Group performance status; ALT: albumin, alanine aminotransferase; AST: aspartate aminotransferase; ALP: alkaline phosphatase; γ-GT: gamma glutamyl transpeptidase; AFP: alpha-fetoprotein; RFA: radiofrequency ablation; PEI: percutaneous ethanol injection; TACE: transarterial chemoembolization.

**Table 2:** Tumor staging information of 196 patients with unresectable HCC.

Staging system	Patients (%)
**Sixth edition TNM**	
1–2	8
3	75
4	17
**Okuda stage**	
I	21
II	62
III	17
**BCLC**	
A	2
B	9
C	57
D	32
**CLIP score**	
0	2
1	19
2	26
3	27
4	16
5	5
6	5
**CUPI classification**	
Low risk group	36
Intermediate risk group	50
High risk group	14
**JIS score**	
0	3
1	14
2	27
3	31
4	16
5	9
**CIS score**	
0	11
1	28
2	29
3	18
4	7
5	7

Abbreviations: HCC: hepatocellular carcinoma; BCLC: Barcelona Clinic Liver Cancer; CLIP: Cancer of the Liver Italian Program; JIS: Japan Integrated Staging; CUPI: Chinese University Prognostic Index; CIS: China integrated score.

They were staged by using each of the seven different staging systems mentioned above. Most of the patients were in the intermediate-advanced stages of disease (75% stage III and 17% stage IV on the basis of TNM sixth edition and 62% stage II and 17% stage III on the basis of Okuda stage) with preserved liver function status (60% Child- Pugh A). HBV was present in 89% of patients. 81% of patients were accompanied with cirrhosis. The overwhelming majority of patients received TACE and best support care.

### Baseline predictors of survival

Independent predictors for overall survival identified through univariate and multivariate analysis are summarized in **Table 3**. Of note, laboratory prognostic factors that were not included in the different staging systems are serum sodium. Other identified prognostic factors included elevated alkaline phosphatase, performance status, tumor size >5 cm, the presence of portal vein invasion, the presence of lymph node metastasis and the presence of distant metastasis. In this patients population, AFP was not related to the survival outcome.

**Table 3:** Independent prognostic factors for overall survival in the 196 patients with unresectalbe HCC according to univariate and multivariate analysis.

Variable	Hazard Ratio for Death	95%CI	*P*
Serum sodium	0.902	0.836–0.931	.000
Alkaline phosphatase	1.002	1.001–1.004	.001
ECOG PS	1.689	1.252–2.277	.001
Tumor size (>5 cm)	1.364	1.107–1.975	.001
Portal vein invasion	2.913	1.900–4.467	.000
Lymph node metastasis	2.032	1.049–3.934	.035
Distant metastasis	2.311	1.305–4.090	.004

Abbreviations: HCC: hepatocellular carcinoma; ECOG PS: Eastern Cooperative Oncology Group performance status.

### Staging system and survival

**Figure 1:**
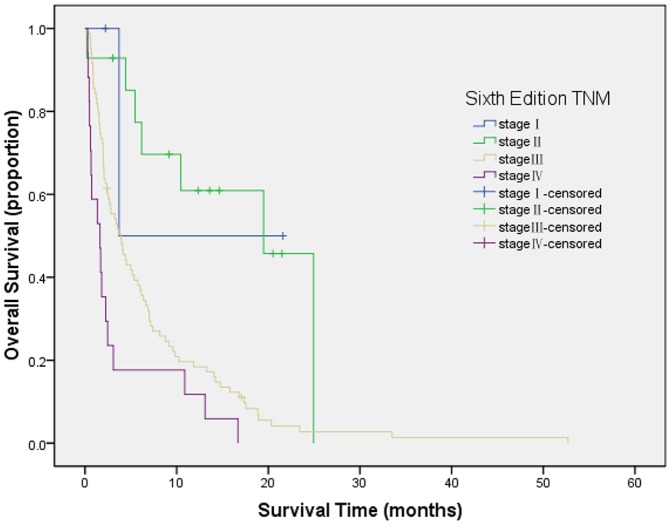
Survival curves for patients with unresectable hepatocellular carcinoma who were stratified according to the TNM sixth edition staging system. Stage I versus II, P = .704; stage II versus III, P = .009; stage III Versus IV, P = .000. The difference between stage I and II was not statistically significant.

Survival curves were generated by Kaplan-Meier method for each of all seven staging systems. Results are summarized as follows from Figure 1 to Figure 7. The Survival curves showed clear different prognostic strata for CUPI and BCLC with statistical significance (log-rank *P*<.001 in all cases). Although some overlapping of survival curves is observed for Okuda, JIS, CLIP, CIS, and TNM sixth edition, overall the difference in survival among different prognostic strata is also statistically significant (log-rank *P*<.001 in both cases).

**Figure 2:**
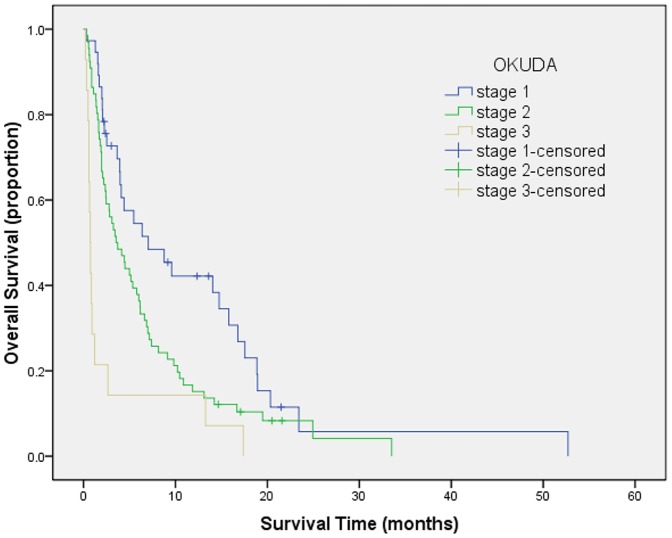
Survival curves for patients with unresectable hepatocellular carcinoma who stratified according to the Okuda staging system. Stage 1 versus 2, P = .015; stage 2 versus 3, P = .010. Statistical difference was noted between any stages.

**Figure 3:**
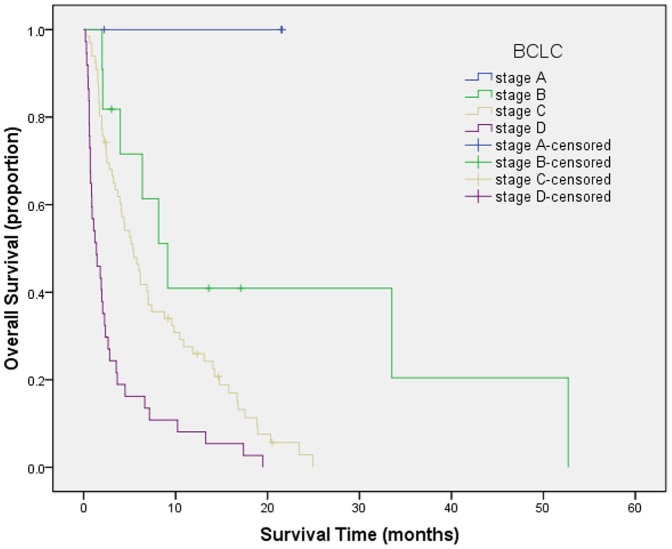
Survival curves for patients with unresectable hepatocellular carcinoma who were stratified according to the Barcelona Clinic Liver Cancer staging system. Stage A versus B, P = .045; stage B versus C, P = .022; stage C versus D, P = .000. All difference between groups was statistically significant.

**Figure 4:**
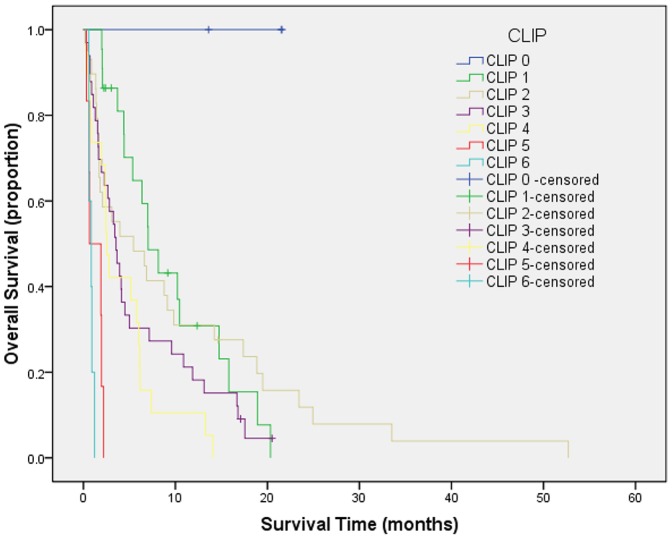
Survival curves for patients with unresectable hepatocellular carcinoma who stratified according to the Cancer of the Liver Italian Program staging system. Score 0 versus 1, P = .050; score 1 versus 2, P = .056; score 2 versus 3, P = .412; score 3 versus 4, P = .518; score 4 versus 5, P = .033; score 5 versus 6, P = .464. No statistical differences were noted between any scores.

**Figure 5:**
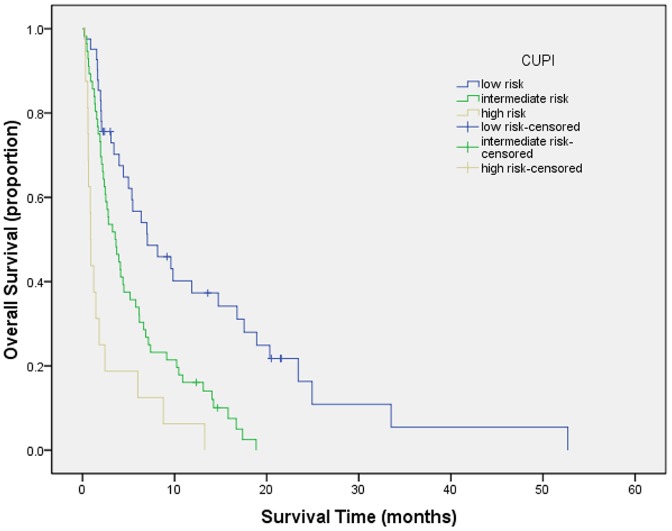
Survival curves for patients with unresectable hepatocellular carcinoma who stratified according to the Chinese University Prognostic Index. Low risk versus intermediate risk, P = .013; intermediate risk versus high risk, P = .000; low risk versus high risk, P = .000. All differences between scores were statistically significant.

**Figure 6:**
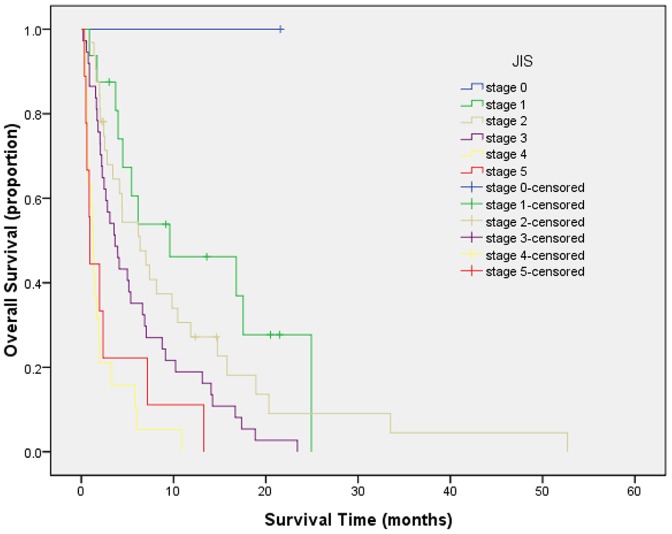
Survival curves for patients with unresectable hepatocellular carcinoma who were stratified according to the Japan Integrated Staging System. Score 0 versus 1, P = .322; score 1 versus 2, P = .655; score 2 versus 3, P = .046; score 3 versus 4, P = .000; score 4 versus 5, P = .980. The difference was significant only between scores of 2 and 3 and between scores of 3 and 4.

**Figure 7:**
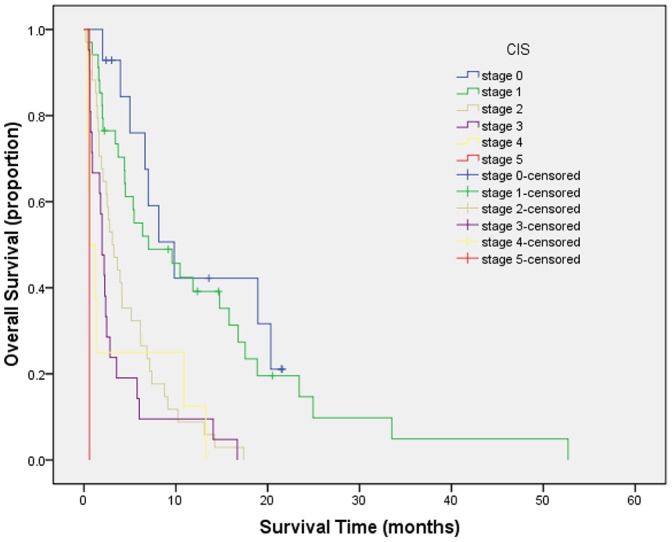
Survival curves for patients with unresectable hepatocellular carcinoma who stratified according to the China Integrated Score. Score 0 versus 1, P = .366; score 1 versus 2, P = .004; score 2 versus 3, P = .056; score 3 versus 4, P = .093; score 4 versus 5, P = .853. The difference was significant only between scores of 1 and 2.

### Ranking of discriminatory ability of staging system

The prognostic ability of the different staging systems was compared through the c-index (**Table 4**). According to the c-index, CUPI (0.746; 95% CI, 0.630 to 0.875) was the first top-ranking staging system, BCLC (0.743; 95% CI, 0.626 to 0.867) was the second top-ranking staging system, and there was statistically significant difference was found among each other (CUPI compared with BCLC, *P*<.01). TNM sixth edition (c-index, 0.734), Okuda (c-index, 0.732), JIS (c-index, 0.728), CIS (c-index, 0.628), and CLIP (c-index, 0.618) were all significantly less valuable than CUPI (*P*<.001).

**Table 4:** Ranking of staging systems in patients with unresectable HCC by using c-index.

Rank	System	C- index	95% CI
1	CUPI	0.746	0.630 to 0.875
2	BCLC	0.743	0.626 to 0.867
3	TNM sixth edition	0.734	0.614 to 0.862
4	Okuda	0.732	0.610 to 0.858
5	JIS	0.728	0.603 to 0.841
6	CIS	0.628	0.504 to 0.751
7	CLIP	0.618	0.482 to 0.754

Abbreviations: HCC: hepatocellular carcinoma; BCLC: Barcelona Clinic Liver Cancer; CLIP: Cancer of the Liver Italian Program; JIS: Japan Integrated Staging; CUPI: Chinese University Prognostic Index; CIS: China integrated score.

### Improvement of staging systems

Addition of the independent prognostic variables of serum sodium improved the discriminatory ability of CUPI with a new c-index of 0.790 compared with 0.746 (bootstrap validated).

## Discussion

At present, the optimal staging system for HCC is still under intense debate. Design of a tumor prognostic model relies on the identification of individual variables that can predict survival of patients with HCC. In this study, we selected the patient population with unresectable HCC which HBV infection is the predominant etiology to study prognostic factors. The study showed that the extent of tumor (tumor size, portal vein involvement, lymph node metastasis and extrahepatic metastasis), hepatic function (Serum sodium, alkaline phosphatase), and overall well-being of the patient (performance status) were independent baseline predictors. So the seven key factors affecting unresectable HCC prognosis were important in our cohort of patients, while AFP is not risk factor in patients with unrescetable HCC in our study.

Performance status had been shown to be an independent prognostic factor of survival in a study on the natural history of untreated HCC [19]. It is strongly associated with survival in HCC patients.

A total of 81% of our patients had underlying cirrhosis, so it is not surprising that survival was related to liver function. We found that individual laboratory tests, serum sodium and alkaline phosphatase, can be well predict survival compared with Child-Pugh classification. Recent studies found that, addition of serum sodium, MELD revealed a better predictor of survival in patients waiting for a liver transplantation [20], [21].

Portal vein involvement, lymph node metastasis and extrahepatic metastasis had been found to be poor prognostic variables in multiple studies [22]–[26]. Portal venous thrombosis can lead to complications of portal hypertension. Furthermore, portal vein involvement is one of the major modes of metastasis of HCC, leading to progression of disease due to direct invasion of adjacent organs/tissues and distant metastasis. Tumor burden had also been shown to be an important prognostic factor, but the cutoff used has varied from a tumor involving more than 50% of the liver to 5 cm diameter of the largest nodule.

Using Kaplan-Meier analysis, we showed that the CUPI system of all seven tumor staging systems currently in use for HCC was the best at discriminating survival of patients in different stages. We believe that the CUPI system had the best prognostication in our cohort because it included the independent predictors of survival we identified: performance status, measure of hepatic function (serum sodium and alkaline phosphatase), and tumor stage (size, portal vein thrombosis, lymph node metastasis, distant metastasis). The superiority of the CUPI was also demonstrated in a recent prospective study of 595 Chinese patients of which HBV infection is the predominant etiology and the median survival was 6.6 months [7].

In addition, CUPI included AFP, which had no prognostic value in our cohort because only two cases had an AFP level of less than 20 ng/mL, and 58% had an AFP level of 500 ng/mL or more.

In our cohort, BCLC system was the second top-ranking staging system. It is the most comprehensive staging system available. Now it has been viewed “as the standard classification that is used for trial design and clinical management of patients with HCC” on the basis of a commentary report [27], supported by two prospective validation [16], [28]. BCLC is designed with an ability to provide therapeutic options for patients at different stages of disease. It also included performance status, measures of liver function, and tumor staging. The BCLC classification on the basis of empirical synthesis of the preceding three aspects and treatment modalities scores well with intermediate to advanced disease in our study.

CLIP is also a commonly used staging system for patients with HCC. The CLIP system has been externally validated in Korean [11], Taiwanese [12], French [13], Japanese [29], American [14], German [15] cohorts.

However, a study of 4525 patients in Japan had reached opposite conclusions that stratification ability and prognostic predictive power of the JIS score were much better than that of the CLIP score [9].

Similarly, Marrero et al reported that CLIP was able to discriminate survival of patients with stage 0 from those with stages 4, 5, and 6. However, it could not differentiate patients with stages 1, 2, and 3 [16]. In our study, CLIP did not score well, because we were studying a specific niche of 71.5% patients with unresectable HCC who fall under the stage 1, 2, 3 categories in CLIP, which limits its discriminatory abilities.

CIS was a new staging system proposed in 2010 [10]. CIS parameters include the hepatic function as defined by Child-Pugh classification(0–2), the tumor extent as defined by adjusted TNM stage(0–2), and serum level of AFP (≤400 or >400 µg/L; 0–1). Adjust TNM stage can be understood as follows: TNM stage≤III (uninodular or multinodular limited in a single lobe, 0), stage IVa (multinodular with multiple liver lobes or vein involvement or invasion through peritoneal tissues, 1) and IVb (distant metastasis, 2) [10]. The CIS score is calculated by summing up each individual score of three items.

We showed that the Okuda, JIS, CIS and the TNM sixth edition systems were not predictive of survival in our cohort, because TNM sixth edition only includes extent of tumor, and Okuda, JIS, CIS included only a limited assessment of tumor extent and a limited assessment of liver function.

There are several limitations in our study. This is a retrospective and single-center study. So the results may not apply to patients with unresectable HCC in other countries. However, the strengths of our study are the complete data in a large number of patients and long follow-up period. And the epidemiological characteristics of our cohort are concordant with that reported in other studies of Chinese patients with HCC [6], [7].

Many studies comparing staging systems in HCC have revealed different ranking of staging systems [11]–[17]. However, these studies included patients with early to advanced stages, or only early to intermediate stage, or only advance stage of HCC, whereas our studied population included patients with intermediate to advanced disease that is not amenable to radical treatment.

Patients with intermediate to advanced HCC have distinct clinical characteristics, tumor extent, and residual liver function. This study reveals once again that for different stages of HCC, the relevance of certain prognostic factors and usefulness of staging systems might vary.

In conclusion, our study shows that performance status, measures of hepatic function (Serum sodium, alkaline phosphatase), tumor characteristics (size, presence of portal vein thrombosis, presence of lymph node metastasis and presence of distant metastasis) are predictors of survival in Chinese patients with unresectable HCC. We show that among the seven prognostic staging systems available for HCC, CUPI provided the best independent prediction of survival in Chinese patients with unresectable HCC. The second top-ranking staging system is BCLC. CLIP has limited discriminatory abilities in this population, and we do not recommend its use in this cohort. The superior performance of CUPI may be related to the fact that it includes the same characteristics that had been identified as independent predictive variables in our cohort. HCC is detected at a late tumor stage in patients with advanced cirrhosis in China. It may be that the CUPI grading system performs best in patients with advanced HCC and advanced cirrhosis and portal hypertension. This would make this system less suitable for European, North American and Japanese patient cohorts. Furthermore, prospective and multicenter validation is required to determine if CUPI system can be accurately used to stratify patients in clinical trials and to help direct medical care.
